# Supplementary material for: Understanding the high-order network plasticity mechanisms of ultrasound neuromodulation
Source: PLoS Comput Biol. 2025 Oct 6;21(10):e1013514. doi: 10.1371/journal.pcbi.1013514 (PMC12500120; doi:10.1371/journal.pcbi.1013514)
Supplement: S1 Text — (DOCX) [file pcbi.1013514.s008.docx]

**Data acquisition**

During both sessions, MRI scans were conducted using a General Electric 3 Tesla scanner equipped with a 48- channel head coil. For the first session: A T1-weighted magnetization-prepared rapid gradient echo (MPRAGE) sequence was performed with a repetition time (TR) of 2.282 seconds, an echo time (TE) of 2.96 milliseconds, an inversion time (TI) of 800 milliseconds, a flip angle (FA) of 8◦, and a field of view (FOV) of 256 × 256 mm, covering 180 slices with 1mm3 isotropic voxels. A Zero Echo Time (ZTE) sequence was also acquired with a TR of 0.531 seconds, a TE of 0.016 milliseconds, an FOV of 256 x 256 mm, and 176 slices with voxel dimensions of 1.016 x 1.016 x 1 mm3. Shimming was performed using two echoes with a TE of 4.54 milliseconds, an FOV of 240 x 240 mm, and 32 slices with voxel dimensions of 3.75 x 3.75 x 5.8 mm3 in the right-to-left direction. Additionally, a Diffusion-Weighted Imaging (DWI) sequence was acquired with a TR of 4.6 seconds, a TE of 90 milliseconds, 63 slices with voxel dimensions of 2.019 x 2.09 x 2 mm3, in the right-to-left direction, and b-values of 0, 300, 1000, and 2000 s/mm2 with 0, 10, 50, and 50 directions, respectively. A 14-minute resting-state functional MRI (rs-fMRI) sequence was also obtained with a TR of 1.4 seconds, a TE of 35 milliseconds, an FA of 68◦, a FOV of 212 × 212 mm, and 88 interleaved slices with 2mm3 isotropic voxels and no slice gap, using a multiband acceleration factor of 3. Cardiac and respiratory data were recorded during this sequence, which was performed with the participants’ eyes open.

For the second session: A 42-minute resting-state functional MRI sequence was conducted with the same parameters as the initial visit—TR of 1.4 seconds, TE of 35 milliseconds, FA of 68◦, FOV of 212 × 212 mm, 88 interleaved slices with 2mm3 isotropic voxels, no slice gap, and a multiband acceleration factor of 3—along with cardiac and respiratory data registration. Additionally, a T1-weighted magnetization-prepared rapid gradient echo (MPRAGE) sequence was repeated with the same parameters as during the first visit: TR of 2.282 seconds, TE of 2.96 milliseconds, TI of 800 milliseconds, FA of 8◦, and FOV of 256 × 256 mm, covering 180 slices with 1mm3 isotropic voxels.

**Ultrasound stimulation**

We applied the stimulation using a NeuroFUS PRO TPO-203 with the four-element CTX-500-4CH transducer (Sonic Concepts, Brainbox Ltd., Cardiff, United Kingdom). The theta-burst TUS protocol was used [2,3] as follows: central frequency=500kHz, pulse duration=20ms, pulse repetition interval=200ms (i.e., duty cycle=10%), total duration=80sec. The ISPPA was set at 54.51W/cm2, following the safety guidelines [1]. To ensure effective coupling between the ultrasound transducer and the participant’s head, ultrasound transmission gel was applied directly to the head, followed by application to the transducer face, and careful manual removal of any air bubbles.

**fMRI pre-processing**

The preprocessing pipeline consists of first removing signals from the ventricles, white matter (removed with Freesurfer), and cardiac and respiratory artifacts. Then, we despike the data and applied the afni proc.py script, including the following steps: blocks (discarding the first two volumes), tshift, align (with the minimum outlier volume), tlrc, volreg, mask, scale, regress (including motion). Finally, we applied a 4mm smooth. The time-series were parcellated into 84 regions using the Desikan-Killiany atlas, including subcortical areas.

**Diffusion MRI pre-processing**

DTI data were preprocessed using the standard approach provided by MRtrix, including denoising, removal of Gibbs artifacts, FSL preprocessing, B1 field inhomogeneity correction, and unsupervised estimation of brain tissues’ multi-shell multi-tissue fiber orientation distributions. Subsequently, ten million tracts were estimated using a probabilistic approach before being reduced to 1 million after tcksift correction for streamline densities. Analogous to the time-series, the DTI matrices were parcellated using the Desikan-Killiany atlas, including subcortical areas, resulting in matrices with dimensions R84×84. This procedure derived the structural connectivity, measured as the average number of streamlines, and the distance, measured as the average length of streamlines between two regions. Three images were excluded due to excessive motion.

# Bibliography

1. Jean-Francois Aubry, David Attali, Mark Schafer, Elsa Fouragnan, Charles Caskey, Robert Chen, Ghazaleh Darmani, Ellen J Bubrick, Jerome Sallet, Christopher Butler, Charlotte Stagg, Miriam Klein-Flugge, Seung- Schik Yoo, Brad Treeby, Lennart Verhagen, and Kim Butts Pauly. Itrusst consensus on biophysical safety for transcranial ultrasonic stimulation. *arXiv*:2311.05359
2. Siti N. Yaakub, Tristan A. White, Jamie Roberts, Eleanor Martin, Lennart Verhagen, Charlotte J. Stagg, Stephen Hall, and Elsa F. Fouragnan. Transcranial focused ultrasound-mediated neurochemical and functional connectivity changes in deep cortical regions in humans. *Nature Communications*, 14:5318, 9 2023.
3. Ke Zeng, Ghazaleh Darmani, Anton Fomenko, Xue Xia, Stephanie Tran, Jean Francois Nankoo, Yazan Shamli Oghli, Yanqiu Wang, Andres M. Lozano, and Robert Chen. Induction of human motor cortex plasticity by theta burst transcranial ultrasound stimulation. *Annals of Neurology*, 91:238–252, 2 2022.
